# Three unrelated and unexpected amino acids determine the susceptibility of the interface cysteine to a sulfhydryl reagent in the triosephosphate isomerases of two trypanosomes

**DOI:** 10.1371/journal.pone.0189525

**Published:** 2018-01-17

**Authors:** Selma Díaz-Mazariegos, Nallely Cabrera, Ruy Perez-Montfort

**Affiliations:** Departamento de Bioquímica y Biología Estructural, Instituto de Fisiología Celular, Universidad Nacional Autónoma de México, Ciudad de México, México; Universidade Nova de Lisboa Instituto de Tecnologia Quimica e Biologica, PORTUGAL

## Abstract

Proteins with great sequence similarity usually have similar structure, function and other physicochemical properties. But in many cases, one or more of the physicochemical or functional characteristics differ, sometimes very considerably, among these homologous proteins. To better understand how critical amino acids determine quantitative properties of function in proteins, the responsible residues must be located and identified. This can be difficult to achieve, particularly in cases where multiple amino acids are involved. In this work, two triosephosphate isomerases with very high similarity from two related human parasites were used to address one such problem. We demonstrate that a seventy-fold difference in the reactivity of an interface cysteine to the sulfhydryl reagent methylmethane sulfonate in these two enzymes depends on three amino acids located far away from this critical residue and which could not have been predicted using other current methods. Starting from previous observations with chimeric proteins involving these two triosephosphate isomerases, we developed a strategy involving additive mutant enzymes and selected site directed mutants to locate and identify the three amino acids. These three residues seem to induce changes in the interface cysteine in reactivity by increasing (or decreasing) its apparent pKa. Some enzymes with four to seven mutations also exhibited altered reactivity. This study completes a strategy for identifying key residues in the sequences of proteins that can have applications in future protein structure-function studies.

## Introduction

Sequence similarity searching is the most common method to identify homologous sequences. It is generally assumed that proteins with similar sequences will usually be homologous (share a common ancestry) and also have a similar function [1]. This is particularly so when the sequence identity is very high [2–4]. One would expect two nearly identical enzyme sequences to be very similar in their three dimensional structures, their catalytic functions and also in their physicochemical characteristics, as well as their susceptibility to chemical and physical inactivating agents. Yet, although in general terms this is true in many cases, very frequently there also exist great differences in the magnitude of the efficiency of the function, the stability of the proteins to physical and chemical denaturants, or in their susceptibility to different types of inactivating agents. These can vary in ranges of orders of magnitude, thus permitting, with the appropriate manipulation, the transformation of specific properties of the proteins within the bounds of these differences and perhaps, sometimes, extending them to greater extremes. An example of this phenomenon are the triosephosphate isomerases (TIMs) from *Trypanosoma brucei* and *T*. *cruzi*. These TIMs have 73.9% identity and a sequence similarity of 92.4%. As expected, their three-dimensional structures superimpose with an RMSD of 0.96 Å and both have an identical catalytic site in each monomer formed by residues K13, H95 and E167 (based on the numbering for the sequence of TIM from *T*. *brucei* (TbTIM) which will be used throughout this article). TIM is the prototype of the (β/α)_8_ barrel fold family of proteins and is active only as a homodimer. However, these extremely similar proteins have some outstanding differences in several functional properties and their behavior to physico-chemical agents and inactivating molecules. For example, their velocity and extent of reactivation from guanidine chloride unfolded monomers [5,6], their susceptibility to digestion with subtilisin [7], and quantitative differences in their susceptibility to several low molecular weight agents [8], and in particular to sulfhydryl reagents [9–13].

A question that arises from observing these large differences is: which amino acid(s) in the sequence is (are) responsible for fine-tuning these differences in behavior?

Some time ago, we started developing a strategy that would help us find an answer to this question. In the work of Garcia-Torres et al. [14] we divided the sequence of TIM into eight interchangeable modular regions. We then progressively grafted different portions of TIM from one trypanosome (*T*. *brucei*) (TbTIM) into the equivalent region of the TIM of the other trypanosome (*T*. *cruzi*) (TcTIM), with the different trait. Following this procedure, we found out which part of the enzyme participated in the expression of the feature that interested us, namely the 70-fold difference in susceptibility to inactivation of the enzymatic activity with the thiol reagent methylmethane thiosulfonate (MMTS). Here it is important to point out that previous work has established that the reaction of MMTS with both enzymes is the unique interface cysteine (Cys) which is in position 14 or 15 of the sequences of TbTIM and TcTIM, respectively [9–13,15–17]. We found that the change in susceptibility to MMTS was due to two regions (regions 1 and 4) of the enzyme that are not in contact with the interface cysteines, which react initially with the thiol reagent, destabilizing and monomerizing the homo-oligomeric protein and causing its inactivation. These two regions are not connected to each other, neither in their primary or tertiary structure. Comparing the sequences of TbTIM and TcTIM, region 1 has thirteen different residues and region 4 has five different residues, respectively. Our conclusion in that work was that, of a total of sixty-five differences between the sequences of the two trypanosomes, we could assign the high or low susceptibility to inactivation in the presence of MMTS to the eighteen different amino acids located in regions 1 and 4. But at that time, the question remained if those eighteen residues were all necessary to produce TbTIM-like or TcTIM-like behaviors.

This work answers the question by refining the strategy, introducing first “additive mutations” [6] and then multiple site directed mutagenesis of the wild type (WT) TIMs.

Our whole general approach to find the key amino acids is ordered and systematic. It initially involves progressive grafting of different large portions of one of these two proteins to an equivalent region of the other protein. This allows the identification of the region, or regions, that contribute to the quantitative differences under investigation. Once these have been established the participation of all the different amino acids contained in the region, or regions responsible for the difference, is explored by “additive” mutants. As in the initial identification of regions with chimeric enzymes, only the residues identified by “additive” mutagenesis that have a positive effect are taken into account. Finally, systematic site-directed mutagenesis of the identified amino acids reveals their role in the occurrence, control and extent of the differences in the behavior that is being studied. Although our scheme involves the testing and analysis of numerous mutant enzymes, we prefer it to random mutagenesis approaches. With this procedure, a comparatively small and limited number of enzymes are monitored by direct experimental results and no other considerations need to be taken into account.

It is important to point out, that this same strategy has also worked in identifying the residues which are responsible for the differences in reactivation velocity and efficiency of TbTIM and TcTIM [6], thus broadening the scope of its application.

## Results

### The amino acids in regions 1 and 4 of TbTIM confer resistance to the inactivation of chimeric enzyme TbTIM 1, 4; TcTIM 2–3, 5–8, using the sulfhydryl reagent MMTS

In our previous work, we showed that the chimeric enzyme TbTIM 1, 4; TcTIM 2–3, 5–8, which has a sequence containing 92.8% of the amino acids of WT TcTIM, has an inactivation profile that is comparable, and even slightly more resistant, than WT TbTIM when exposed to the sulfhydryl reagent MMTS [14]. This implies that within the 18 amino acids that are different in these two regions, either all, or a subset of them, account for the differences in the susceptibility to MMTS in TcTIM and TbTIM. What is clear from that work is that one or more amino acids from both regions 1 and 4 are necessary to change the behavior of the enzymes to inactivation by MMTS.

The strategy we devised to find out which, and how many, amino acids are involved conferring MMTS-susceptibility to these enzymes, consisted in constructing additive mutants from previously reported chimeric proteins (see Materials and Methods) and also site-directed mutagenesis of selected amino acids from regions 1 and 4 in enzymes with the sequences of WT TbTIM and WT TcTIM, respectively.

**Fig 1:**
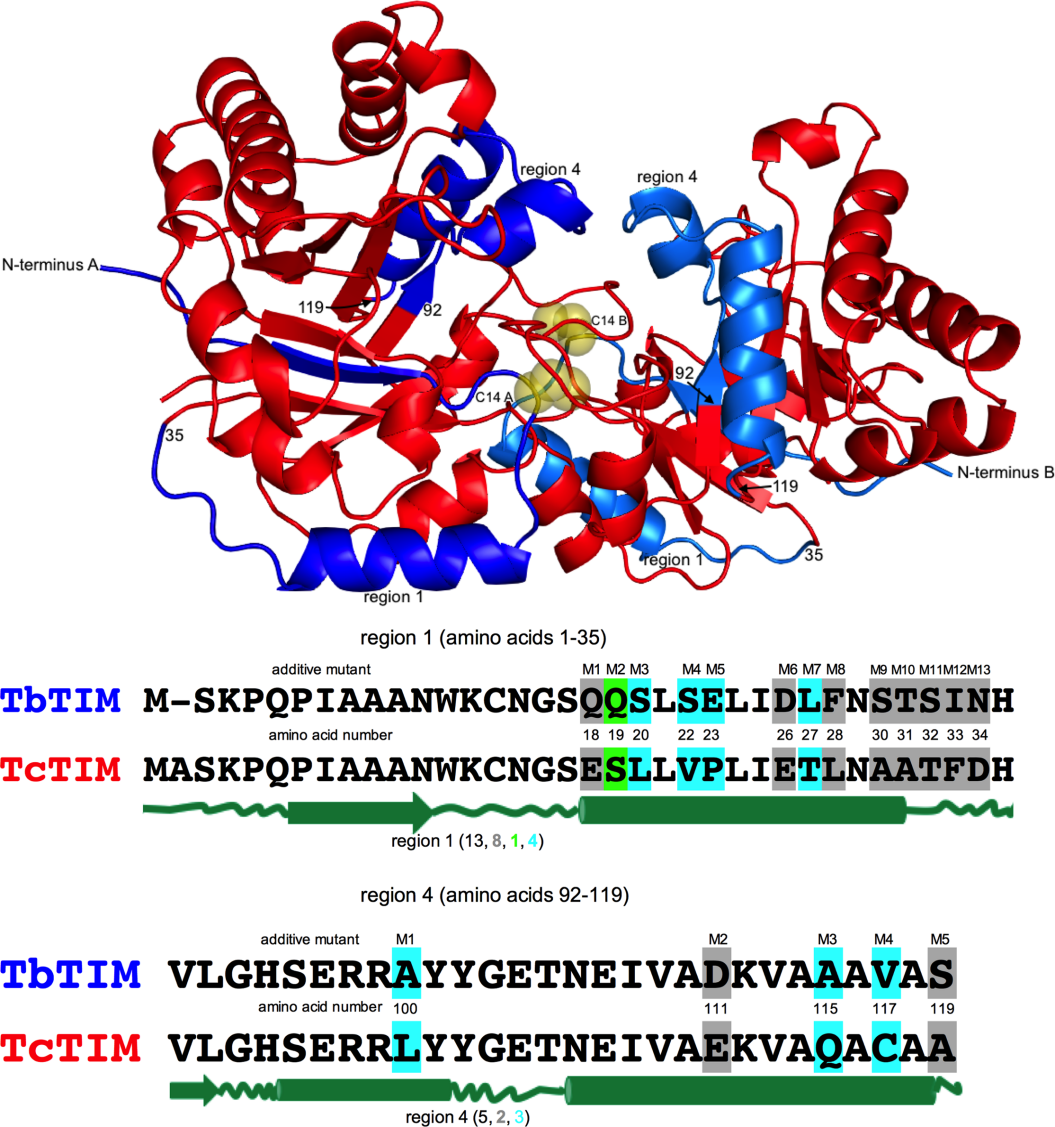
Location of regions 1 and 4 in a ribbon diagram of the structure of WT TbTIM and aligned sequences of regions 1 and 4 of WT TbTIM and WT TcTIM. The ribbon diagram of the structure of 5TIM in the PDB database, corresponding to a dimer of WT TbTIM, is shown in red. Regions 1 and 4 are shown in blue and marine blue for monomers A and B, respectively. The n-terminus of each monomer and the position of amino acids 35, 92 and 119 are indicated by the corresponding text or number, sometimes using an arrow, on the diagram. The interface cysteines of both monomers are shown as yellow spheres. In the alignments, the differences in the amino acids are highlighted as conservative (similar size and polarity) in grey, semiconservative (similar polarity) in green, and without similarity in cyan. Secondary structure elements are shown below as dark green lines (loops), arrows (beta sheets) and barrels (alpha helixes).

The sequences of WT TbTIM and WT TcTIM have 13 differences in region 1 and 5 differences in region 4 (Fig 1).

### Susceptibility to inactivation with MMTS of the additive mutants of region 1

**Fig 2:**
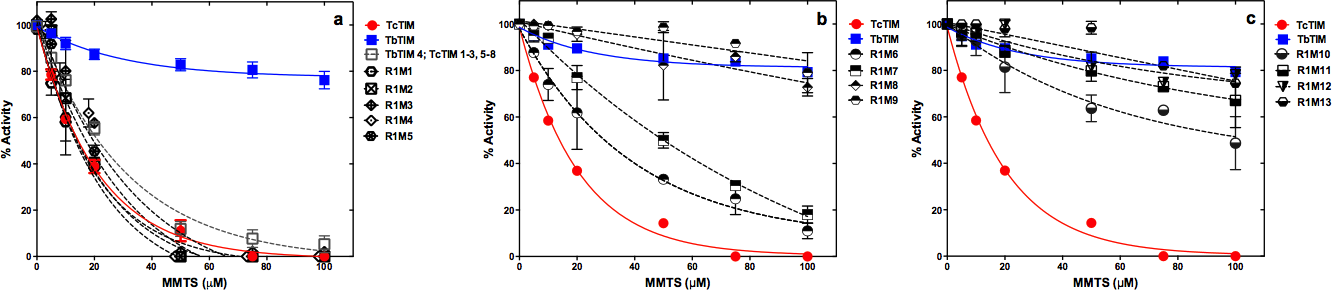
Effect of MMTS on WT TbTIM, WT TcTIM and on different additive mutants of region 1. Enzymes were incubated at a concentration of 250 μg/mL in 100 mM TEA, 10 mM EDTA pH 7.4 and 5–100 μM MMTS for 2 h at 25°C. At that time, the activity of the samples was determined, including a sample without MMTS to calculate the percentage of remaining activity. Panel a) effect of MMTS on WT TcTIM, WT TbTIM and R1M1, R1M2, R1M3, R1M4, R1M5 and TbTIM 4; TcTIM 1–3, 5–8. Panel b) effect of MMTS on WT TcTIM, WT TbTIM and R1M6, R1M7, R1M8 and R1M9. Panel c) effect of MMTS on WT TcTIM, WT TbTIM and R1M10, R1M11, R1M12 and R1M13. Assays were performed independently three times.

To identify which amino acids in region 1 are important in determining the susceptibility of TbTIM and TcTIM to inactivation by MMTS, chimeric enzyme TbTIM 4; TcTIM 1–3, 5–8 was chosen as the starting point, since this protein inactivates almost like WT TcTIM (see Fig 2C in [14] and Fig 2A), and an increase in resistance to inactivation was to be expected. In the inactivation curves of the thirteen additive mutants of region 1 (Fig 2) we observed that the amino acids in positions 18, 19, 20, 22 and 23 (which are Q, Q, S, S and E in the sequence of TbTIM, and include additive mutants R1M1, R1M2, R1M3, R1M4 and R1M5, respectively) have practically no effect on the inactivation pattern of chimeric enzyme TbTIM 4; TcTIM 1–3, 5–8 (see Fig 2A).

When additive mutant R1M6 (which has the additional mutation of E26D) was tested, the resistance to inactivation to 100 μM MMTS was increased to 11% and the form of the curve showed increased resistance to inactivation in all other concentrations of MMTS studied (Fig 2B). Additive mutant R1M7, which introduces mutation T27L, yields an enzyme that is somewhat more resistant than the preceding one (Fig 2B). At this point, the importance of the amino acids in positions 26 and 27 is not apparent yet, but it will be made clear in the section of further analysis of site-directed mutagenesis of selected amino acids from regions 1 and 4 in enzymes with the sequences of WT TbTIM and WT TcTIM.

The importance of the amino acid in position 28 was immediately evident observing the behavior of additive mutant R1M8 (with mutation L28F), since this enzyme resists inactivation with 100 μM MMTS, resembling WT TbTIM with an activity of 72.8% (Fig 2B). The effect of the substitution of a L for a F and of a F for a L in position 28 of the sequence of TcTIM and TbTIM, respectively, will also be described in the section of further analysis of site-directed mutagenesis of selected amino acids from regions 1 and 4 in enzymes with the sequences of WT TbTIM and WT TcTIM.

Additive mutant R1M9 (A30S) did not show any significant change of behavior, with respect to the previous additive mutant (Fig 2B), and additive mutant R1M10 (A31T) even increased the susceptibility of the resulting enzyme to inactivation with MMTS (decreasing the activity to 48% in the presence of 100 μM MMTS) (Fig 2C).

Here it is important to point out that additive mutations could produce positive, negative or neutral effects on the susceptibility pattern, when compared with the additive mutant that preceded it. As will become clear further on for the development of our strategy, we only chose to consider and use the mutations that exhibited a positive effect in causing resistance to the inactivating effects of MMTS.

Additive mutant R1M11 with mutation T32S did show an increased resistance to inactivation with MMTS with respect to the preceding mutant (67% in the presence of 100 μM MMTS) (Fig 2C).

Finally, additive mutants R1M12 and R1M13, with mutations F33I and D34N respectively, were neutral with respect to additive mutant R1M11 and exhibited essentially the same inactivation pattern in the presence of MMTS seen for WT TbTIM (Fig 2C).

### Susceptibility to inactivation with MMTS of the additive mutants of region 4

**Fig 3:**
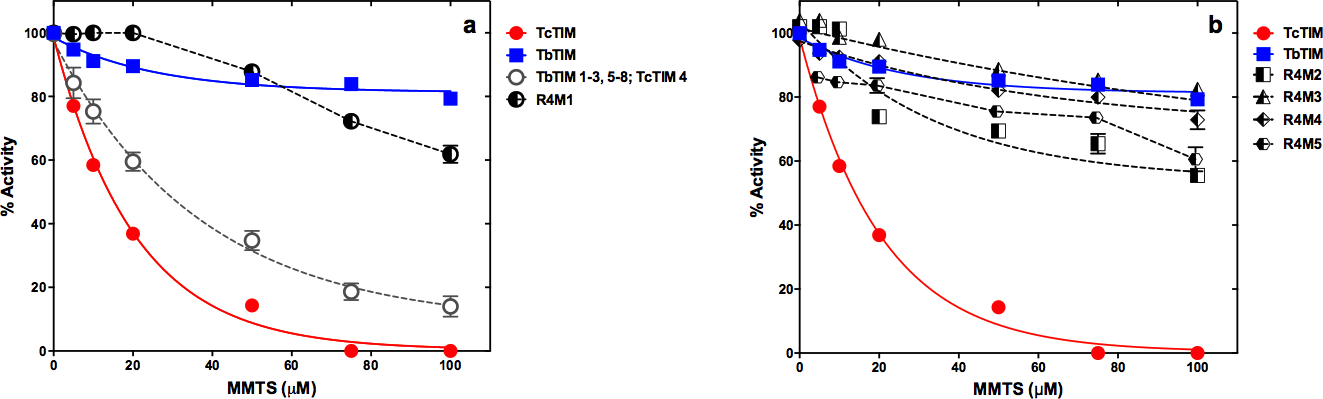
Effect of MMTS on WT TbTIM, WT TcTIM and on different additive mutants of region 4. Enzymes were incubated at a concentration of 250 μg/mL in 100 mM TEA, 10 mM EDTA pH 7.4 and 5–100 μM MMTS for 2 h at 25°C. At that time, the activity of the samples was determined, including a sample without MMTS to calculate the percentage of remaining activity. Panel a) effect of MMTS on WT TcTIM, WT TbTIM, R4M1 and TbTIM 1–3, 5–8; TcTIM 4. Panel b) effect of MMTS on WT TcTIM, WT TbTIM and R4M2, R4M3, R4M4 and R4M5. Assays were performed independently three times.

The five amino acids in region 4 were tested by using the chimeric enzyme TbTIM 1–3, 5–8; TcTIM 4 as the starting point, since this protein, which has 98% of the sequence of TbTIM, inactivates following a similar pattern to WT TcTIM (see Fig 2C in [14] and Fig 3A). So, once again, an increase in resistance to inactivation was to be expected. In the inactivation curves of the five additive mutants of region 4 (Fig 3) we observed that the amino acid in position 100 has a very important effect in conferring resistance to MMTS. This additive mutant is R4M1, which has the mutation L100A. The residual activity shown by additive mutant R4M1 at a concentration of 100 μM MMTS is 62%, which is approximately 20% lower than that of WT TbTIM under the same conditions, but, the resistance to inactivation of additive mutant R4M1 at concentrations of MMTS of 50 μM or lower is even higher than that of WT TbTIM (Fig 3A). So, it seems that the alanine at position 100 in the sequence of TbTIM has an important role in the resistance of this protein to inactivation by low concentrations of MMTS. In contrast, additive mutant R4M2 showed an increase in susceptibility to inactivation with MMTS, producing a negative effect with respect to the previous mutant (Fig 3B).

The next additive mutant R4M3, with mutation Q115A, which again introduces an alanine, had another positive effect in increasing the resistance of the enzyme to inactivation with MMTS with a pattern that is slightly more susceptible to the reagent than WT TbTIM at all the concentrations of MMTS studied (Fig 3B).

Additive mutation R4M4 was neutral in its effect to the preceding mutation and the last additive mutation R4M5 had a slightly negative effect, returning to a susceptibility pattern that is very similar to that of the first additive mutant R4M1 (Fig 3B).

### Site directed mutagenesis of selected amino acids of regions 1 and 4 from the sequence of TbTIM onto TcTIM

**Fig 4:**
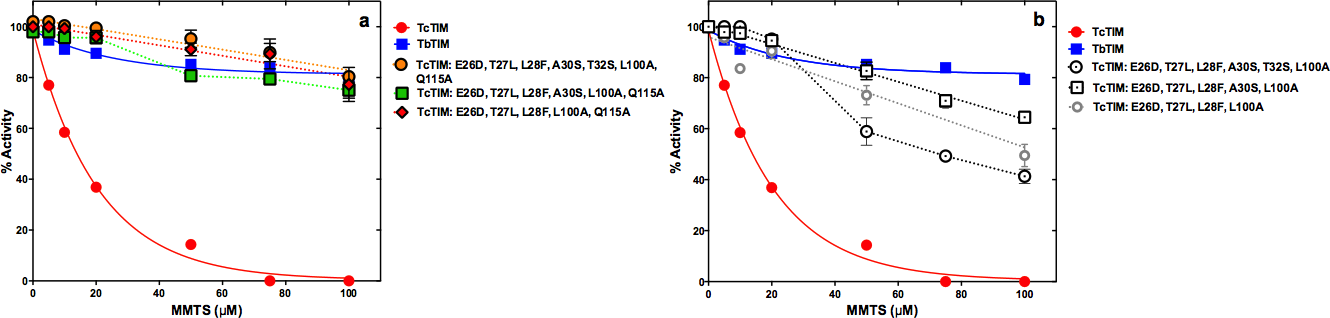
Effect of MMTS on WT TbTIM, WT TcTIM and on different site directed mutants of selected amino acids of regions 1 and 4 of the sequence of WT TbTIM onto WT TcTIM. Enzymes were incubated at a concentration of 250 μg/mL in 100 mM TEA, 10 mM EDTA pH 7.4 and 5–100 μM MMTS for 2 h at 25°C. At that time, the activity of the samples was determined, including a sample without MMTS to calculate the percentage of remaining activity. Panel a) effect of MMTS on WT TcTIM, WT TbTIM and TcTIM: E26D, T27L, L28F, A30S, T32S, L100A, Q115A, TcTIM: E26D, T27L, L28F, A30S, L100A, Q115A and TcTIM: E26D, T27L, L28F, L100A, Q115A. Panel b) effect of MMTS on WT TcTIM, WT TbTIM and TcTIM: E26D, T27L, L28F, A30S, T32S, L100A; TcTIM: E26D, T27L, L28F, A30S, L100A and TcTIM: E26D, T27L, L28F, L100A. Assays were performed independently three times.

Judging from our previous results with the additive mutants of regions 1 and 4, we could conclude that the amino acids that have a positive effect of conferring resistance to inactivation with MMTS are five in positions 26, 27, 28, 30 and 32, of region 1, and two in positions 100 and 115, of region 4. Thus, to produce a TcTIM with the inactivation susceptibility pattern of TbTIM, we initially constructed the site directed mutant TcTIM: E26D, T27L, L28F, A30S, T32S, L100A, Q115A. As expected, and can be seen in Fig 4A, the enzyme with seven mutations showed a similar and even slightly better resistance pattern to the inactivation with MMTS than WT TbTIM, particularly at concentrations lower than 100 μM.

Since the positive effect of mutations A30S and T32S, which corresponded to additive mutants R1M9 and R1M11, respectively, had not been too great compared with their individual previous mutants, we decided to prepare the following site directed mutants, to explore if all five amino acids of region 1 were necessary to change the susceptibility response of the first mutant that contained seven mutations. Thus, site directed mutants TcTIM: E26D, T27L, L28F, A30S, L100A, Q115A and TcTIM: E26D, T27L, L28F, L100A, Q115A were prepared and subjected to inactivation with MMTS. Fig 4A shows that both enzymes, with six and five mutations, still show a susceptibility pattern that is essentially the same as that of WT TbTIM. The curve of the six-fold mutant was almost completely superimposable to the curve of WT TbTIM, while the curve of the five-fold mutant was superimposable to the seven-fold mutant, again, with a slightly increased resistance pattern at low concentrations of the sulfhydryl reagent.

Once it was established that two mutations in region 1 with relatively small positive effects on the susceptibility pattern could be omitted and the resulting enzyme still shows a behavior like that of WT TbTIM being inactivated with MMTS, we turned our attention to the residue in position 115 of region 4. This was because the change of Q for A in additive mutant R4M3 had been positive but not as great as that for the change of L for A in additive mutant R4M1.

Nevertheless, we decided to produce three site directed mutants with six, five and four mutations, deliberately omitting to change the amino acid in position 115.

These site directed mutants were: TcTIM: E26D, T27L, L28F, A30S, T32S, L100A; TcTIM: E26D, T27L, L28F, A30S, L100A and TcTIM: E26D, T27L, L28F, L100A, respectively.

As can be seen in Fig 4B, all these mutant enzymes were more susceptible than WT TbTIM to inactivation by MMTS, particularly at concentrations of 50 μM and above. There was no evident correlation between the number of mutants and the shape of the inactivation curve. Thus, the alanine in position 115 of the sequence of TbTIM was important for the resistance to inactivation by higher concentrations of MMTS.

### Further analysis

Starting from the five-fold mutant TcTIM: E26D, T27L, L28F, L100A, Q115A, with the susceptibility pattern comparable to WT TbTIM, we decided to establish if we could further reduce the number of changes in the sequence of TcTIM, and still retain the behavior with increased resistance to inactivation by MMTS. Knowing the importance of an alanine in position 115 of region 4 to achieve TbTIM-like resistance at higher concentrations of MMTS, this residue was always included in subsequent experiments. We therefore prepared three site directed mutants with four mutations which were: TcTIM: T27L, L28F, L100A, Q115A; TcTIM: E26D, L28F, L100A, Q115A and TcTIM: E26D, T27L, L100A, Q115A.

**Fig 5:**
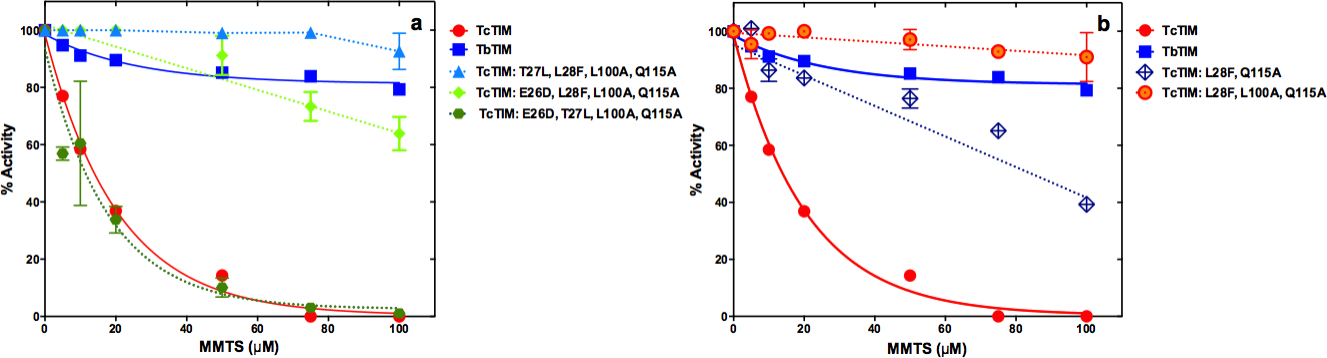
Effect of MMTS on WT TbTIM, WT TcTIM and on different site directed mutants of selected amino acids of regions 1 and 4 of the sequence of WT TbTIM onto WT TcTIM. Enzymes were incubated at a concentration of 250 μg/mL in 100 mM TEA, 10 mM EDTA pH 7.4 and 5–100 μM MMTS for 2 h at 25°C. At that time, the activity of the samples was determined, including a sample without MMTS to calculate the percentage of remaining activity. Panel a) effect of MMTS on WT TcTIM, WT TbTIM and TcTIM: T27L, L28F, L100A, Q115A; TcTIM: E26D, L28F, L100A, Q115A and TcTIM: E26D, T27L, L100A, Q115A. Panel b) effect of MMTS on WT TcTIM, WT TbTIM, TcTIM: L28F, L100A, Q115A and TcTIM: L28F, Q115A. Assays were performed independently three times.

The inactivation curves at different concentrations of MMTS can be seen in Fig 5A. From the results, it is evident that the amino acid in position 28 is fundamental in determining the inactivation profile of the corresponding site directed mutant enzyme. A phenylalanine will give an enzyme that resists inactivation in a manner similar to WT TbTIM, and a leucine will increase susceptibility to the levels of WT TcTIM.

To explore the influence of residues in positions 26 and 27, a triple site directed mutant was prepared: TcTIM: L28F, L100A, Q115A. The inactivation curve, seen in Fig 5B, shows a profile that indicates more resistance of the mutant enzyme to inactivation with all concentrations of MMTS than WT TbTIM.

So, we now had a mutant of TcTIM with 98.8% of its sequence, which had an inactivation profile like that of TbTIM. The only amino acid, whose role had not been tested, was that in position 100 of region 4. Consequently, we produced double site directed mutant TcTIM: L28F, Q115A. As can be seen in Fig 5B the inactivation curve of this mutant shows an intermediate behavior between WT TbTIM and WT TcTIM.

These experiments suggested that a minimum of three mutations, one in region 1 and two in region 4, are sufficient and necessary to change the inactivation profile of WT TcTIM to that of WT TbTIM.

### Confirmation with site directed mutants of TbTIM

**Fig 6:**
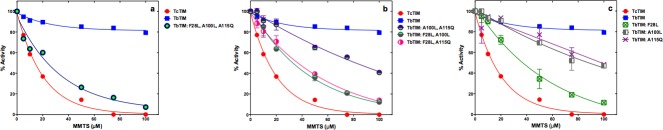
Effect of MMTS on WT TbTIM, WT TcTIM and on different site directed mutants of selected amino acids of regions 1 and 4 on the sequence of WT TbTIM. Enzymes were incubated at a concentration of 250 μg/mL in 100 mM TEA, 10 mM EDTA pH 7.4 and 5–100 μM MMTS for 2 h at 25°C. At that time, the activity of the samples was determined, including a sample without MMTS to calculate the percentage of remaining activity. Panel a) effect of MMTS on WT TcTIM, WT TbTIM and TbTIM: F28L, A100L, A115Q. Panel b) effect of MMTS on WT TcTIM, WT TbTIM and TbTIM: A100L, A115Q, TbTIM: F28L, A100L and TbTIM: F28L, A115Q. Panel c) effect of MMTS on WT TcTIM, WT TbTIM and TbTIM: F28L, TbTIM: A100L and TbTIM: A115Q. Assays were performed independently three times.

Complete or partial confirmation of the results shown previously could be obtained if site directed mutants of WT TbTIM in residues in positions 28, 100 and 115 showed and increased susceptibility to inactivation by MMTS, resembling, or equal to, that of WT TcTIM. Thus, the site directed mutant TbTIM: F28L, A100L, A115Q was prepared and tested. The corresponding results can be seen in Fig 6A where the mutant, with 98.8% of the sequence of WT TbTIM, exhibited an inactivation pattern that resembles that of WT TcTIM, being just as susceptible at concentrations of MMTS of 10 μM or lower and slightly more resistant at concentrations above 20 μM.

The contribution of the individual or the three combinations of pairs of mutated amino acids was also investigated. As can be seen in Fig 6B and 6C the observation made previously with TcTIM that the phenylalanine in position 28 is fundamental to confer resistance to inactivation with MMTS is confirmed in the single and double site directed mutants of TbTIM.

Thus, our results show that the inactivation profiles of WT TbTIM and WT TcTIM depend mainly on three residues: one located in position 28 (of region 1) and two located in positions 100 and 115 (of region 4), completely confirming our previous results with the chimeric enzyme TbTIM 1, 4; TcTIM 2–3, 5–8 [14].

### The pKa of the interface Cys14 is regulated by amino acid in position 28 of region 1 and amino acids in positions 100 and 115 of region 4

Although the mechanism by which the difference in susceptibility to inactivation with MMTS occurs is not clear, we have previously reported that the reactivity of the interface Cys14 in TbTIM and TcTIM depends on the pKa of its thiol group [18]. In our most recent determination it was 10.53 for WT TbTIM and 9.27 for WT TcTIM [14]. Table 1 shows that the thiol group of Cys14 of the triple site directed mutant TcTIM: L28F, L100A, Q115A has a very similar pKa to that of WT TbTIM (10.69) and that the pKa of Cys14 of triple mutant TbTIM: F28L, A100L, A115Q is very similar to that of WT TcTIM (9.23).

**Table 1:** pKa values of Cys 14/15 in WT TbTIM and WT TcTIM and some mutant enzymes.

Enzyme or mutant	pKa of Cys14
WT TbTIM	10.56 ± 0.22
WT TcTIM	9.24 ± 0.04
TcTIM: L28F, L100A, Q115A	10.69 ± 0.01
TbTIM: F28L, A100L, A115Q	9.23 ± 0.03

Based on these results, the predicted ratio of protonated/deprotonated sulfhydryl groups at pH 7.4 are 1445, 69, 1950, and 67.6 for WT TbTIM, WT TcTIM, TcTIM: L28F, L100A, Q115A and TbTIM: F28L, A100L, A115Q, respectively. It can be calculated that there are over 20 times more predicted protonated sulfhydryl groups in the proteins with a Cys with low reactivity to MMTS than in the more susceptible ones. Thus, part of the explanation for a greater susceptibility of Cys 14 in WT TcTIM and TbTIM: F28L, A100L, A115Q is due to this higher ratio because the reactivity of MMTS with the protonated thiol group is immensely lower than with the thiolate anion [19].

## Discussion

Our work shows that it is possible to systematically identify the amino acids in a sequence that are responsible for a given property of a protein. Here, we have extended and refined our experimental method of using the same protein from two evolutionarily closely related organisms to identify the regions relevant for a given function [14]. Like our initial approach, the new method again cannot, and does not predict, which mutations would affect the catalytic properties and the susceptibility of the interface Cys to MMTS. It is not biased by structural, evolutionary or hypothetical considerations of the possible, or theoretical, importance of certain amino acids for the function, stability or other properties of the protein, but gives the answer directly from the experimental results.

We think there are several noteworthy insights to be learned from our results. As can be seen in S1 Table (in the supplementary material) basically all chimeric proteins, additive mutants, and site directed mutants had catalytic properties that are comparable to those of the WT enzymes. As already pointed out previously, this is an expected result since, although of the three catalytic amino acids, K13 is in region 1 and H95 is in region 4, the mutations did not affect their properties nor those of other residues that are important for their function. E167 is in region 6, which is far removed from the regions where the mutations were made, thus, no influence of this residue was expected.

From our previous work, we already anticipated that the properties of the interface Cys would not change gradually or continuously, but would respond in a more discontinuous manner. This turned out to be the case. But the answer to the question of the minimum number of residues involved in MMTS susceptibility yielded an interesting answer. On the one hand, our results clearly suggest that a minimum of three residues (in position 28 of region 1 and positions 100 and 115 of region 4) are necessary for a WT TcTIM to change its susceptibility to MMTS inactivation to one like WT TbTIM (Fig 5B), and that these same residues will change the susceptibility of WT TbTIM almost to that of a TcTIM (Fig 6A). Thus, these are the crucial residues, because no double or single mutant can change completely the susceptibility pattern (Fig 6B and 6C). On the other hand, our results also show that other fourfold, fivefold, sixfold and sevenfold mutants of WT TcTIM (Figs 4A and 5A) also have the decreased susceptibility to MMTS shown by WT TbTIM. What is the significance of this?

Random mutagenesis methods usually rely on (rapidly) finding one protein, which shows the altered function or stability characteristics, and then analyze the mutated amino acid or amino acids that produced the change, and, if possible, what the reasons were for it. But, this might be just one of multiple possible mutants that have the altered behavior. In this case these would be mutants TcTIM: E26D, T27L, L28F, A30S, T32S, L100A, Q115A; TcTIM: E26D, T27L, L28F, A30S, L100A, Q115A; TcTIM: E26D, T27L, L28F, L100A, Q115A; TcTIM: T27L, L28F, L100A, Q115A and TcTIM: L28F, L100A, Q115A. Depending on whether it is the minimal number of changes, or some larger number, which still produces the differences, the validation that one has the minimum of mutations to produce the altered behavior will be different. In any case, a systematic approach like the one shown in this work, should be useful to prove and understand that, in many cases, the change in behavior is not due to only one mutation, and also what the importance of each individual amino acid is, when there is a synergic cooperation to produce the altered characteristics and/or a change of function in a protein.

In our opinion, to better understand the role of each amino acid for either, particular physicochemical characteristics, or the function of a protein, a systematic analysis using mutagenesis like the one described in Garcia-Torres et al. [14] and in this work, will identify all the responsible residues even if, and particularly when, they cannot be inferred or predicted by other methods of analysis of the protein.

## Materials and methods

### Design of the genes of different mutant enzymes

This work is a continuation of the work of García-Torres et al. [14], and thus, we used some of the chimeric proteins produced for that investigation. In that article a central conclusion was that the chimeric enzyme TbTIM 1,4;TcTIM 2,3, 5–8 (called TcTIM 2,3, 5–8 in that publication) had an equal or slightly less pronounced susceptibility pattern to inactivation with MMTS than WT TbTIM. The sequence of TbTIM 1,4;TcTIM 2,3,5–8 has eighteen different amino acids from that of WT TcTIM.

To find out which of the thirteen different residues of region 1 or the five different residues of region 4 were involved, we designed the strategy of the “additive mutants”. For the additive mutants of region 1 we started out with chimeric protein TbTIM 4;TcTIM 1–3, 5–8 as the template, and progressively mutated the first different amino acid in region 1, and then the first and second different amino acids in region 1, and then the first, second and third, and so on, until all thirteen different amino acids were “additively” mutated. In this work, the nomenclature for the additive mutants has the form RXMX, where R is the region in which the mutation occurs and M is the number of additive mutants it contains. Therefore, mutant R1M12 has twelve mutations in region 1 and mutant R4M1 has one mutation in region 4 (see S2 Table).

Besides the additive mutants, a series of single and multiple site directed mutant enzymes were produced, to test the importance of individual or combinations of amino acids in the susceptibility or resistance to inactivation in both, the sequences of WT TbTIM and WT TcTIM to the inactivation with MMTS. For this, DNA sequences X03921 and U53867 for TbTIM and TcTIM, respectively, were used to produce all site directed mutant enzymes. From TcTIM a total of eighteen and for TbTIM a total of eight site-directed mutants were produced. Their names are as follows: the sequence on which the site directed mutant was made (either TbTIM or TcTIM) and then the original amino acid, then the position in the sequence and then the mutant amino acid. Thus, TcTIM: E26D, T27L, L28F, A30S, L100A, Q115A is a site directed mutant of WT TcTIM with four mutations in region 1 and two mutations in region 4 in positions 26, 27, 28 and 30 and 100 and 115, respectively.

The site directed mutants, the templates and the oligonucleotides used to produce them are shown in S3 Table in the Supplementary Material.

### Expression and purification of the proteins

All genes were cloned into the pET- 3a expression plasmid using the *Nde*-I and *Bam*HI restriction sites. Every gene was completely sequenced and transformed into BL21(DE3)pLysS cells (Novagen, Madison WI).

Bacteria containing the plasmids with each of the genes were grown in one liter of Luria Bertani medium supplemented with 100 μg/mL ampicillin and were incubated at 37°C. Once the cell cultures reached an A600 nm = 0.8, a final concentration of 1 mM isopropyl -b-D thiogalactopyranoside was used for induction and the bacteria were incubated 12 h more at 30°C before harvesting them.

Harvested bacteria were centrifuged for 20 min at 6400 × *g* and resuspended in 40 ml of lysis buffer (100 mM MES, 1 mM DTT, 0.5 mM EDTA, 0.2 mM PMSF, 300 mM NaCl, pH 6.3) [20]. Each suspension was sonicated at a potency of 5 W for 5 times 40 sec with 1 min rest between each cycle. The sonicated suspensions were centrifuged at 110 660 × *g* for 40 min. The supernatant of each chimeric enzyme or mutant was diluted until the final concentration of NaCl was 20 mM and was then applied to a SP Sepharose Fast flow column that had been previously equilibrated with 50 mM MES pH 6.3. The protein was eluted using a NaCl linear gradient 50 mM MES, NaCl 0–500 mM pH 6.3. Crystalline ammonium sulfate was gradually added up to 70% (w/v) saturation at 4°C under agitation to the fractions containing TIM. This suspension was further agitated for 12 h and then centrifuged for 20 min at 23 000 × *g*. The precipitate was resuspended into 10 ml of 100 mM triethanolamine (TEA), 10 mM EDTA, pH 7.4. Enough ammonium sulfate was added to have a final concentration of 2.2 M.

The protein was then applied to a hydrophobic interaction column of butyl toyopearl that had been previously been equilibrated with 100 mM TEA, 10 mM EDTA and 2.2 M ammonium sulfate. The protein was eluted with a linear gradient of 2.2 to 0 M of ammonium sulfate. The fractions containing TIM were pooled and concentrated. Protein concentration was determined at 280 nm, using an extinction coefficient ε = 34 950 M^-1^ cm^-1^. The extinction coefficients for all the mutants were calculated from their amino acid sequence and the online program ProtParam from Expasy. Also, SDS-PAGE with 16% acrylamide gels was used as a control of the purification procedure for all mutants.

### Determination of catalytic activity

Enzyme activity was measured at 25°C following the conversion of DL-glyceraldehyde 3-phosphate (D,L-GAP) to dihydroxyacetone phosphate using a-glycerolphosphate dehydrogenase (α-GDH) as coupling enzyme [14]. The oxidation of NADH was monitored at 340 nm, and the reaction mixture had 10 mM TEA, 10 mM EDTA, 1 mM GAP, 0.2 mM NADH and 20 μg/mL α-GDH. The reaction was started by adding 5 ng/mL of the corresponding protein [21].

### Calculation of the kinetic parameters

To calculate kinetic parameters, GAP concentration was varied between 0.04 and 3 mM, and the data were adjusted to the Michaelis–Menten model using non-linear regression to calculate K_m_ and V_max_. The catalytic constant k_cat_ and the catalytic efficiency k_cat_/K_m_ were also calculated from these data [15].

### Inactivation assays with MMTS

WT enzymes, as well as all mutants, at a concentration of 250 mg/mL were incubated with concentrations of 5–100 μM of MMTS in a buffer containing 100 mM TEA, 10 mM EDTA, pH 7.4 for 2 h at 25°C. At this time, the mixtures were diluted and an aliquot of the dilution was withdrawn to measure activity at a concentration of 5 ng/mL of reaction mixture in a Cary 60 spectrophotometer from Agilent Technologies (Santa Clara, CA, USA). The activity data are reported as percentage of residual activity, taking the activity of each corresponding enzyme in the absence of MMTS as 100% [14]. Graphs with the data were made using Prism GraphPad version 6.0. Whenever possible, the data of the graphs was adjusted using nonlinear regression. On those curves that did not adjust, lines were drawn joining the points to guide the eye.

#### Determination of the pKa of the interface Cys

The pKa of the interface Cys of WT TbTIM, WT Tc TIM and of site directed mutants TcTIM: L28F, L100A, Q115A; and TbTIM: F28L, A100L, A115Q, was determined as described in reference [18] with some modifications. Briefly, the enzymes were incubated for 1 min at a concentration of 250 μg/mL in 100 mM TEA and 10 mM EDTA adjusted to the desired pH (7.0, 7.4, 7.8, 8.0, 8.4, 8.8, 9.0, 9.2 and 9.5); MMTS at a concentration of 80 μM was also added. The residual catalytic activity was measured at 340 nm in the spectrophotometer as described before. The apparent pKa of the interface Cys was determined from plots of ln of percent remaining activity versus pH. The data were fitted to a model derived from the Henderson-Hasselbach equation:

$$ln\left(\%\;activity\right)=(Y_{i}+Y_{h}\times 10^{pKa-pH})/\left(1+10^{pKa-pH}\right)$$

where Y_i_ and Y_h_ represent the initial and final activities, respectively.

## Supporting information

S1 Table Kinetic parameters of WT TbTIM, WT Tc TIM and additive mutants and mutants obtained by site directed mutagenesis. All data shown are the means of three independent determinations.
(DOCX)

S2 Table Strategy for the production of the additive mutants of regions 1 and 4. The amino acids highlighted in grey, green or cyan were changed from the amino acid present in the sequence of WT TcTIM to the one present in the sequence of WT TbTIM.
(DOCX)

S3 Table Oligonucleotides used for the additive mutagenesis of regions 1 and 4 and the production of selected site directed mutants WT TbTIM and WT TcTIM. (DOCX)
