# Supplementary material for: Three unrelated and unexpected amino acids determine the susceptibility of the interface cysteine to a sulfhydryl reagent in the triosephosphate isomerases of two trypanosomes
Source: PLoS One. 2018 Jan 17;13(1):e0189525. doi: 10.1371/journal.pone.0189525 (PMC5771576; doi:10.1371/journal.pone.0189525)
Supplement: S1 Table — All data shown are the means of three independent determinations. (DOCX) [file pone.0189525.s001.docx]

S1 Table

**Specific activities of wild type TbTIM and TcTIM and eighteen additive mutants of Regions 1 and 4**

| **Enzyme** | **Specific activity (µmol/(min mg)^-1^** |
| --- | --- |
| TcTIM | 4041.8 ± 1.51 |
| TbTIM | 5508.03 ± 1.85 |
| R1M1 | 2539.12 ± 3.03 |
| R1M2 | 3796.94 ± 4.54 |
| R1M3 | 3825.29 ± 2.69 |
| R1M4 | 3274.38 ± 1.15 |
| R1M5 | 3729.90 ± 6.06 |
| R1M6 | 4329.04 ± 3.65 |
| R1M7 | 5310.82 ± 3.56 |
| R1M8 | 3832.79 ± 3.41 |
| R1M9 | 3705.25 ± 3.01 |
| R1M10 | 4539.12 ± 1.51 |
| R1M11 | 3425.50 ± 3.03 |
| R1M12 | 4396.57 ± 2.73 |
| R1M13 | 4805.39 ± 6.71 |
| R4M1 | 4659.57 ± 1.74 |
| R4M2 | 3920.36 ± 2.36 |
| R4M3 | 3789.91 ± 1.81 |
| R4M4 | 3477.16 ± 4.54 |
| R4M5 | 4226.58 ± 1.51 |
| TcTIM: E26D, T27L, L28F, A30S, T32S, L100A, Q115A | 3537.31± 2.33 |
| TcTIM: E26D, T27L, L28F, L100A, Q115A | 5670.97 ± 1.51 |
| TcTIM: E26D, L28F, L100A, Q115A | 4692.07 ± 4.81 |
| TcTIM: L28F, L100A, Q115A | 4959.35 ± 0.52 |
| TbTIM: F28L, A100L, A115Q | 4204.80 ± 2.23 |

**Kinetic constants of wild type TbTIM and TcTIM and three site directed mutants of TcTIM**

| **Enzyme** | **Vmax μmol (min mg)^-1^** | **Km (mM)** | **Kcat (min^-1^)** | **Kcat/Km (M^-1^s^-1^)** |
| --- | --- | --- | --- | --- |
| TbTIM | 5666 | 0.1511 | 3.09x10^5^ | 3.4x10^7^ |
| TcTIM | 4829 | 0.1988 | 2.72x10^5^ | 2.2x10^7^ |
| TcTIM: E26D, T27L, L28F, A30S, T32S, L100A, Q115A | 5311 | 0.246 | 2.90x10^5^ | 1.9x10^7^ |
| TcTIM: E26D, T27L, L28F, L100A, Q115A | 5420 | 0.1505 | 8.02x10^5^ | 8.8x10^7^ |
| TcTIM: T27L, L100A, Q115A | 4204 | 0.5330 | 2.29x10^5^ | 7.1x10^6^ |
