# Supplementary material for: Three unrelated and unexpected amino acids determine the susceptibility of the interface cysteine to a sulfhydryl reagent in the triosephosphate isomerases of two trypanosomes
Source: PLoS One. 2018 Jan 17;13(1):e0189525. doi: 10.1371/journal.pone.0189525 (PMC5771576; doi:10.1371/journal.pone.0189525)
Supplement: S2 Table — The amino acids highlighted in grey, green or cyan were changed from the amino acid present in the sequence of WT TcTIM to the one present in the sequence of WT TbTIM. (DOCX) [file pone.0189525.s002.docx]

S2 Table

**Strategy for the production of the additive mutants of regions 1 and 4**

| **Additive mutant** | **TbTIM region** | **TcTIM regions** | **highlighted mutation(s)** |
| --- | --- | --- | --- |
| R1M1 | 4 | 1-3 and 5-8 | **TbTIM NGSQQSLSELIDLFNSTSINH**  **TcTIM NGSESLLVPLIETLNAATFDH** |
| R1M2 | 4 | 1-3 and 5-8 | **TbTIM NGSQQSLSELIDLFNSTSINH**  **TcTIM NGSESLLVPLIETLNAATFDH** |
| R1M3 | 4 | 1-3 and 5-8 | **TbTIM NGSQQSLSELIDLFNSTSINH**  **TcTIM NGSESLLVPLIETLNAATFDH** |
| R1M4 | 4 | 1-3 and 5-8 | **TbTIM NGSQQSLSELIDLFNSTSINH**  **TcTIM NGSESLLVPLIETLNAATFDH** |
| R1M5 | 4 | 1-3 and 5-8 | **TbTIM NGSQQSLSELIDLFNSTSINH**  **TcTIM NGSESLLVPLIETLNAATFDH** |
| R1M6 | 4 | 1-3 and 5-8 | **TbTIM NGSQQSLSELIDLFNSTSINH**  **TcTIM NGSESLLVPLIETLNAATFDH** |
| R1M7 | 4 | 1-3 and 5-8 | **TbTIM NGSQQSLSELIDLFNSTSINH**  **TcTIM NGSESLLVPLIETLNAATFDH** |
| R1M8 | 4 | 1-3 and 5-8 | **TbTIM NGSQQSLSELIDLFNSTSINH**  **TcTIM NGSESLLVPLIETLNAATFDH** |
| R1M9 | 4 | 1-3 and 5-8 | **TbTIM NGSQQSLSELIDLFNSTSINH**  **TcTIM NGSESLLVPLIETLNAATFDH** |
| R1M10 | 4 | 1-3 and 5-8 | **TbTIM NGSQQSLSELIDLFNSTSINH**  **TcTIM NGSESLLVPLIETLNAATFDH** |
| R1M11 | 4 | 1-3 and 5-8 | **TbTIM NGSQQSLSELIDLFNSTSINH**  **TcTIM NGSESLLVPLIETLNAATFDH** |
| R1M12 | 4 | 1-3 and 5-8 | **TbTIM NGSQQSLSELIDLFNSTSINH**  **TcTIM NGSESLLVPLIETLNAATFDH** |
| R1M13 | 4 | 1-3 and 5-8 | **TbTIM NGSQQSLSELIDLFNSTSINH**  **TcTIM NGSESLLVPLIETLNAATFDH** |

| Additive mutant | TbTIM regions | TcTIM region | highlighted mutation(s) |
| --- | --- | --- | --- |
| R4M1 | 1-3 and 5-8 | 4 | **TbTIM VLGHSERRAYYGETNEIVADKVAAAVAS**  **TcTIM VLGHSERRLYYGETNEIVAEKVAQACAA** |
| R4M2 | 1-3 and 5-8 | 4 | **TbTIM VLGHSERRAYYGETNEIVADKVAAAVAS**  **TcTIM VLGHSERRLYYGETNEIVAEKVAQACAA** |
| R4M3 | 1-3 and 5-8 | 4 | **TbTIM VLGHSERRAYYGETNEIVADKVAAAVAS**  **TcTIM VLGHSERRLYYGETNEIVAEKVAQACAA** |
| R4M4 | 1-3 and 5-8 | 4 | **TbTIM VLGHSERRAYYGETNEIVADKVAAAVAS**  **TcTIM VLGHSERRLYYGETNEIVAEKVAQACAA** |
| R4M5 | 1-3 and 5-8 | 4 | **TbTIM VLGHSERRAYYGETNEIVADKVAAAVAS**  **TcTIM VLGHSERRLYYGETNEIVAEKVAQACAA** |
