# Supplementary material for: Three unrelated and unexpected amino acids determine the susceptibility of the interface cysteine to a sulfhydryl reagent in the triosephosphate isomerases of two trypanosomes
Source: PLoS One. 2018 Jan 17;13(1):e0189525. doi: 10.1371/journal.pone.0189525 (PMC5771576; doi:10.1371/journal.pone.0189525)
Supplement: S3 Table — (DOCX) [file pone.0189525.s003.docx]

S3 Table

**Oligonucleotides**

**Additive mutants of Region 1**

| **Mutant** | **Fw primer** | **Rv primer** |
| --- | --- | --- |
| R1M1 | 5’AAGTGCAACGGCTCCCAGAGTTTGCTTGTACCACTC3’ | 5’GAGTGGTACAAGCAAACTCTGGGAGCCGTTGCACTT3’ |
| R1M2 | 5’TGGAAGTGCAACGGCTCCCAGCAGTTGCTTGTACCACTC3’ | 5’GAGTGGTACAAGCAACTGCTGGGAGCCGTTGCACTTCCA3’ |
| R1M3 | 5’GCAAACTGGAAGTGCAACGGCTCCCAGCAGAGCCTTGTACCA3’ | 5’TGGTACAAGGCTCTGCTGGGAGCCGTTGCACTTCCAGTTTGC3’ |
| R1M4 | 5’AAGTGCAACGGCTCCCAGCAGAGCCTTAGCCCACTCATCGAGACGCTC3’ | 5’GAGCGTCTCGATGAGTGGGCTAAGGCTCTGCTGGGAGCCGTTGCACTT3’ |
| R1M5 | 5’CTGGAAGTGCAACGGCTCCCAGCAGAGCCTTAGCGAACTCATCGAGACGCT3’ | 5’AGCGTCTCGATGAGTTCGCTAAGGCTCTGCTGGGAGCCGTTGCACTTCCAG3’ |
| R1M6 | 5’GAACTCATCGATACGCTCAATGCAGCGACTTTT3’ | 5’AAAAGTCGCTGCATTGAGCGTATCGATGAGTTC3’ |
| R1M7 | 5’GAACTCATCGATCTGCTCAATGCAGCGACTTTTGAT3’ | 5’ATCAAAAGTCGCTGCATTGAGCAGATCGATGAGTTC3’ |
| R1M8 | 5’GAACTCATCGATCTGTTTAATGCAGCGACT3’ | 5’AGTCGCTGCATTAAACAGATCGATGAGTTC3’ |
| R1M9 | 5’ATCGATCTGTTTAATAGCGCGACTTTTGAT3’ | 5’ATCAAAAGTCGCGCTATTAAACAGATCGAT3’ |
| R1M10 | 5’AGCCTTAGCGAACTCATCGATCTGTTTAATAGCACCACTTTTGATCAC3’ | 5’GTGATCAAAAGTGGTGCTATTAAACAGATCGATGAGTTCGCTAAGGCT3’ |
| R1M11 | 5’AGCCTTAGCGAACTCATCGATCTGTTTAATAGCACCAGCTTTGATCACGATGTGCAA3’ | 5’TTGCACATCGTGATCAAAGCTGGTGCTATTAAACAGATCGATGAGTTCGCTAAGGCT3’ |
| R1M12 | 5’AGCCTTAGCGAACTCATCGATCTGTTTAATAGCACCAGCATCGATCACGATGTGCAATGC3’ | 5’GCATTGCACATCGTGATCGATGCTGGTGCTATTAAACAGATCGATGAGTTCGCTAAGGCT3’ |
| R1M13 | 5’AGCCTTAGCGAACTCATCGATCTGTTTAATAGCACCAGCATCAACCACGATGTGCAATGCGTGGTT3’ | 5’AACCACGCATTGCACATCGTGGTTGATGCTGGTGCTATTAAACAGATCGATGAGTTCGCTAAGGCT3’ |

**Additive mutants of Region 4**

| **Mutant** | **Fw primer** | **Rv primer** |
| --- | --- | --- |
| R4M1 | 5’GGTCACTCCGAGCGCCGCGCGTACTACGGCGAAACGAACGAAATCGTT3’ | 5’AACGATTTCGTTCGTTTCGCCGTAGTACGCGCGGCGCTCGGAGTGACC3’ |
| R4M2 | 5’TCCGAGCGCCGCGCGTACTACGGCGAAACGAACGAAATCGTTGCGGATAAGGTGGCGCAGGCC3’ | 5’GGCCTGCGCCACCTTATCCGCAACGATTTCGTTCGTTTCGCCGTAGTACGCGCGGCGCTCGGA3’ |
| R4M3 | 5’GAAACGAACGAAATCGTTGCGGATAAGGTGGCGGCGGCCTGCGCTGCC3’ | 5’GGCAGCGCAGGCCGCCGCCACCTTATCCGCAACGATTTCGTTCGTTTC3’ |
| R4M4 | 5’AACGAAATCGTTGCGGATAAGGTGGCGGCGGCCGTGGCTGCCGGCTTCATGGT3’ | 5’ACCATGAAGCCGGCAGCCACGGCCGCCGCCACCTTATCCGCAACGATTTCGTT3’ |
| R4M5 | 5’GCGGATAAGGTGGCGGCGGCCGTGGCTAGCGGCTTCATGGTTATTGCTTGCATC3’ | 5’GATGCAAGCAATAACCATGAAGCCGCTAGCCACGGCCGCCGCCACCTTATCCGC3’ |

**Site directed mutants of TcTIM**

| **Mutant** | **Fw primer** | **Rv primer** |
| --- | --- | --- |
| TcTIM: E26D, T27L, L28F, A30S, T32S, L100A, Q115A | 5’CTGTTTAATAGCGCGAGCTTTGATCACGATGTGCAATGCGTG3’ | 5’CACGCATTGCACATCGTGATCAAAGCTCGCGCTATTAAACAG3’ |
| TcTIM: E26D, T27L, L28F, A30S, T32S, L100A | 5’CTGTTTAATAGCGCGAGCTTTGATCACGATGTGCAATGCGTG3’ | 5’CACGCATTGCACATCGTGATCAAAGCTCGCGCTATTAAACAG3’ |
| TcTIM: E26D, T27L, L28F, A30S, L100A, Q115A | 5’TTGCTTGTACCACTCATCGATCTGTTTAATAGCGCGACTTTTGATCAC3’ | 5’GTGATCAAAAGTCGCGCTATTAAACAGATCGATGAGTGGTACAAGCAA3’ |
| TcTIM: E26D, T27L, L28F, A30S, L100A | 5’TTGCTTGTACCACTCATCGATCTGTTTAATAGCGCGACTTTTGATCAC3’ | 5’GTGATCAAAAGTCGCGCTATTAAACAGATCGATGAGTGGTACAAGCAA3’ |
| TcTIM: E26D, T27L, L28F, L100A, Q115A | 5’TTGCTTGTACCACTCATCGATCTGTTTAATGCAGCGACTTTTGAT3’ | 5’ATCAAAAGTCGCTGCATTAAACAGATCGATGAGTGGTACAAGCAA3’ |
| TcTIM: E26D, T27L, L28F, L100A | 5’TTGCTTGTACCACTCATCGATCTGTTTAATGCAGCGACTTTTGAT3’ | 5’ATCAAAAGTCGCTGCATTAAACAGATCGATGAGTGGTACAAGCAA3’ |
| TcTIM: T27L, L28F, L100A, Q115A | 5’CTTGTACCACTCATCGAACTGTTTAATGCAGCGACT3’ | 5’AGTCGCTGCATTAAACAGTTCGATGAGTGGTACAAG3’ |
| TcTIM: E26D, L28F, L100A, Q115A | 5’CTTGTACCACTCATCGATACCTTTAATGCAGCGACT3’ | 5’AGTCGCTGCATTAAAGGTATCGATGAGTGGTACAAG3’ |
| TcTIM: E26D, T27L, L100A, Q115A | 5’CTTGTACCACTCATCGATCTGCTGAATGCAGCGACT3’ | 5’AGTCGCTGCATTCAGCAGATCGATGAGTGGTACAAG3’ |
| TcTIM: T27L, L28F, L100A | 5’GCGGAAAAGGTGGCGCAGGCCTGCGCTGCCGGC3’ | 5’GCCGGCAGCGCAGGCCTGCGCCACCTTTTCCGC3’ |
| TcTIM: L28F, L100A, Q115A | 5’GTACCACTCATCGAGACGTTTAATGCAGCGACTTTTGAT3’ | 5’ATCAAAAGTCGCTGCATTAAACGTCTCGATGAGTGGTAC3’ |
| TcTIM: T27L, L100A, Q115A | 5’CCACTCATCGAGCTGCTCAATGCAGCGACTTTT3’ | 5’AAAAGTCGCTGCATTGAGCAGCTCGATGAGTGG3’ |
| TcTIM: L28F, Q115A | 5’CCACTCATCGAGACGTTTAATGCAGCGACTTTT3’ | 5’AAAAGTCGCTGCATTAAACGTCTCGATGAGTGG3’ |
| TcTIM: L28F, L100A | 5’CGTTTCGCCGTAGTACGCACGCCGTTCCGA3’ | 5’TCGGAACGGCGTGCGTACTACGGCGAAACG3’ |
| TcTIM: L100A, Q115A | 5’GTTGCGGAAAAGGTGGCGGCGGCCTGCGCTGCCGGCTTC3’ | 5’GAAGCCGGCAGCGCAGGCCGCCGCCACCTTTTCCGCAAC3’ |
| TcTIM: L28F | 5’CCACTCATCGAGACGTTTAATGCAGCGACTTTT3’ | 5’AAAAGTCGCTGCATTAAACGTCTCGATGAGTGG3’ |
| TcTIM: L100A | 5’CGTTTCGCCGTAGTACGCACGCCGTTCCGA3’ | 5’TCGGAACGGCGTGCGTACTACGGCGAAACG3’ |
| TcTIM: Q115A | 5’GTTGCGGAAAAGGTGGCGGCGGCCTGCGCTGCCGGCTTC3’ | 5’GAAGCCGGCAGCGCAGGCCGCCGCCACCTTTTCCGCAAC3**’** |

**Site directed mutants of TbTIM**

| **Mutant** | **Fw primer** | **Rv primer** |
| --- | --- | --- |
| TbTIM: F28L, A100L, A115Q | 5’CACTCCGAGCGCCGCCTGTACTATGGTGAGACA3’ | 5’TGTCTCACCATAGTACAGGCGGCGCTCGGAGTG3’ |
| TbTIM: A100L, A115Q | 5’GCGGACAAGGTTGCCCAGGCCGTTGCTTCTGGT3’ | 5’ACCAGAAGCAACGGCCTGGGCAACCTTGTCCGC3’ |
| TbTIM: F28L, A115Q | 5’GCGGACAAGGTTGCCCAGGCCGTTGCTTCTGGT3’ | 5’ACCAGAAGCAACGGCCTGGGCAACCTTGTCCGC3’ |
| TbTIM: F28L, A100L | 5’CACTCCGAGCGCCGCCTGTACTATGGTGAGACA3’ | 5’TGTCTCACCATAGTACAGGCGGCGCTCGGAGTG3’ |
| TbTIM: A115Q | 5’GCGGACAAGGTTGCCCAGGCCGTTGCTTCTGGT3’ | 5’ACCAGAAGCAACGGCCTGGGCAACCTTGTCCGC3’ |
| TbTIM: A100L | 5’CACTCCGAGCGCCGCCTGTACTATGGTGAGACA3’ | 5’TGTCTCACCATAGTACAGGCGGCGCTCGGAGTG3’ |
| TbTIM: F28L | 5’GAGCTTATTGATCTGCTGAACTCCACAAGCATC3’ | 5’GATGCTTGTGGAGTTCAGCAGATCAATAAGCTC3’ |
